# Thermoelectric Generators (TEGs) and Renewable-Energy-Integrated Membrane-Based Hybrid Desalination Systems

**DOI:** 10.3390/membranes16050175

**Published:** 2026-05-13

**Authors:** M. Hamza Asif Awan, Ashraf Aly Hassan, Asad Ali Zaidi, Muhammad Asad Javed

**Affiliations:** 1 Department of Mechanical Engineering, School of Mechanical & Manufacturing Engineering (SMME), National University of Sciences and Technology (NUST), H-12, Islamabad 44000, Pakistan; mawan.me23smme@student.nust.edu.pk; 2 Department of Civil and Environmental Engineering, United Arab Emirates University, Al Ain 15551, United Arab Emirates; alyhassan@uaeu.ac.ae; 3 Department of Mechanical Engineering, Faculty of Engineering, Islamic University of Madinah, Medina 42351, Saudi Arabia; sali@iu.edu.sa; 4 Sustainability Research Center, Islamic University of Madinah, Medina 42351, Saudi Arabia

**Keywords:** membrane desalination, thermoelectric generators, hybrid desalination systems, renewable energy, osmosis, low-grade heat recovery

## Abstract

Population growth, industrialization and climate change have placed increasing stress on natural freshwater reserves, making conventional water sources inadequate. Coupled with rising energy constraints and environmental concerns, interest in desalination technologies that can operate more sustainably and efficiently has intensified. Among the available approaches, membrane desalination has gained particular importance because of its modularity, relatively low energy demand, and compatibility with decentralized water treatment. In parallel, thermoelectric devices have emerged as promising components for hybrid desalination systems due to their ability to convert temperature gradients into electricity or provide localized heating and cooling for process enhancement. This article presents a narrative review of thermoelectric integration in desalination systems, with particular emphasis on membrane desalination and membrane-hybrid water treatment configurations powered by renewable-energy or low-grade heat sources. The review examines the role of thermoelectric devices in relation to key membrane-based and hybrid desalination processes, including reverse osmosis, membrane distillation, electrodialysis, nanofiltration, forward osmosis, and selected hybrid systems. Particular attention is given to system configurations, renewable energy coupling pathways, functional roles of thermoelectric devices, water productivity, module output, desalination efficiency, water quality, and economic performance. The reviewed literature indicates that thermoelectric integration can provide meaningful benefits in hybrid desalination, particularly through improved thermal management, enhanced utilization of low-grade heat, and supplementary energy recovery. These opportunities appear especially relevant for thermally driven membrane systems such as membrane distillation and for membrane-hybrid configurations intended for decentralized or renewable-powered applications. However, the available evidence remains highly heterogeneous, with substantial variation in system scale, operating conditions, reporting metrics, and cost assumptions, which limits direct cross-study comparison and broad generalization of performance claims. This review highlights the technical challenges, reporting inconsistencies, and research gaps that currently constrain the practical development of thermoelectric-assisted membrane desalination and outlines future directions for membrane-aligned hybrid desalination research.

## 1. Introduction

Water is an essential part of life. Access to usable clean water remains one of the most critical challenges globally. The United Nations Environment Program (UNEP) states that only one third of the world’s population has sufficient access to fresh water [1]. As a result, drinking water of acceptable quality has become a luxury for many. The total global water reserves are roughly 1.4 billion km^3^ [2]. Of this, 97.5% exists in the oceans as undrinkable water and only the remaining 2.5% is present in the atmosphere, ice caps, glaciers and ground water as fresh water [2]. Of the total, only 0.014% is readily available for human consumption [2]. This imbalance between freshwater availability and demand is further exaggerated by the uneven geographic distribution and seasonal variability of water resources. There is also an increased demand for fresh water in the world due to population growth. However, the demand for fresh water is twice the population growth rate, due to increased demand for fresh water by both the industrial and agricultural sectors [3,4]. It is estimated that by 2030 water consumption will grow by 50% [5].

To meet the ever-increasing demand for fresh water, the first desalination plant was installed in the 1950s [6,7]. The early desalination plants involved thermal processes such as distillation, due to the low cost of fossil fuels (less than $3 for one barrel of oil) [8]. These thermal plants demonstrated the technical feasibility of converting seawater into potable water, even though the costs were high. Over time, though, through technological improvement and research, desalination has been transformed into a globally adapted water supply strategy. In 2020 there were roughly 18,000 desalination plants in the world with an estimated capacity of 99.8 million m^3^·day^−1^ [9]. Today this number is still growing with approximately 21,000 operational plants which provide fresh water to nearly 300 million people [10,11]. The sector’s growth capacity is estimated to be between 6% and 12% yearly [12].

Despite these worldwide adaptations, conventional desalination still remains energy-intensive, leading to high operational costs and adverse environmental footprints. The combustion of fossil fuels also emits large quantities of greenhouse gases, GHGs (i.e., CO, CO_2_, NO_2_, SO_2_ and NO). It is estimated that around 25 kg·m^−3^ of CO_2_ is emitted [13]. Desalination plants powered by on-grid electricity, generated by conventional sources, generate approximately 1.0 kg of CO_2_ per kWh or 3.8 kg CO_2_ per m^3^ of desalinated water. In response to these drawbacks, recent research has focused on developing energy-efficient and sustainable desalination technologies. Incorporating renewable energy in desalination as the primary or secondary energy source is one way of counteracting the production of GHGs. An alternative strategy is to make the systems more efficient, to limit the amount of GHGs produced. In recent years, membrane desalination technologies have become increasingly important in sustainable water treatment because of their modularity, comparatively lower specific energy demand, and suitability for decentralized deployment. Processes such as reverse osmosis, membrane distillation, electrodialysis, nanofiltration, and forward osmosis are now widely recognized as key components of next-generation desalination systems, particularly when coupled with renewable energy or waste-heat resources [14]. Their operational flexibility and compatibility with hybrid energy configurations make membrane processes especially relevant in the search for efficient and scalable desalination strategies.

Furthermore, hybrid systems integrating multiple desalination technologies along with energy recovery are being explored to enhance overall system performance. Combining thermal and membrane desalination methods has been one of the hot topics of research for the last decade [15]. Helal et al. [16] established that a hybrid system consisting of a reverse osmosis (RO) system coupled with a thermal process such as MSF can result in 20–30% of thermal energy being saved as compared to standalone thermal plants. RO systems coupled with ED have been shown to have an energy consumption rate of 7.77 kWh·m^−3^ and a high water recovery rate of 81.1% compared to a water recovery rate of 42.6% for a standalone SWRO system [17]. In addition, zero liquid discharge (ZLD) can be used to process brine to eliminate hazardous chemicals and protect marine life [18]. Such integrated approaches are becoming increasingly important in addressing both water scarcity and climate-related challenges.

In parallel, thermoelectric devices have attracted growing interest for desalination-related applications because of their ability to convert temperature gradients into electricity and, in some configurations, provide localized heating or cooling. These capabilities make thermoelectric integration potentially valuable for hybrid desalination systems in which renewable energy, low-grade heat, or thermal gradients are already present. Beyond direct electricity generation, thermoelectric modules may also contribute to improved thermal management, enhanced use of waste heat, and support for compact system designs. These features are particularly relevant for membrane-based and membrane-hybrid desalination processes where thermal control, modular integration, and energy utilization are important design considerations. Thermoelectric devices offer many advantages such as being maintenance-free and having a long lifetime. On average a typical thermoelectric device can have a service life of over 100,000 h without any degradation [19]. The mean time between failure (MTBF) is more than 20 years for steady-state conditions [20].

Although a number of studies have examined renewable-energy-driven desalination and hybrid desalination systems, the role of thermoelectric devices has often been discussed in a fragmented manner across different process classes and operating scales. In particular, the literature lacks a clearly focused review that interprets thermoelectric integration from the perspective of membrane desalination and membrane-hybrid water treatment systems. As a result, the potential relevance of thermoelectric assistance for membrane processes, especially under renewable-powered or low-grade-heat conditions, remains insufficiently synthesized and critically assessed.

Accordingly, the present article provides a narrative review of thermoelectric integration in desalination systems, with particular emphasis on membrane desalination and membrane-hybrid configurations. The review discusses the main desalination process categories, examines renewable-energy coupling pathways, and evaluates the functional roles of thermoelectric devices in relation to water productivity, thermoelectric output, desalination efficiency, water quality, and economic performance. Special attention is given to the implications of thermoelectric coupling for membrane-based systems and to the research gaps that must be addressed for their practical development. In this way, the review aims to provide a more membrane-aligned perspective on thermoelectric-assisted desalination and to identify directions for future research in sustainable hybrid water treatment.

### Scope of This Review and Relevance to Membrane Desalination

This article presents a narrative review of thermoelectric integration in desalination systems powered by renewable energy or low-grade heat, with particular emphasis on their relevance to membrane desalination and membrane-hybrid water treatment systems. Although desalination technologies span both thermal and membrane-based routes, membrane processes have become increasingly important because of their comparatively lower energy demand, modular design, operational flexibility, and suitability for decentralized water production [21]. In this context, the incorporation of thermoelectric devices offers additional opportunities for process intensification, localized thermal management, auxiliary power generation, and improved utilization of renewable or waste-energy streams.

The reviewed literature includes studies on hybrid and cogenerative desalination systems in which thermoelectric generators (TEGs) or thermoelectric modules (TEMs) are coupled with desalination units to enhance water productivity, improve thermal efficiency, recover low-grade heat, or support auxiliary electrical loads. While non-membrane systems are also discussed, they are included mainly to provide background, establish comparative context, and highlight the broader landscape within which membrane desalination technologies are evolving. Particular attention is given to membrane-related processes such as reverse osmosis (RO), membrane distillation (MD), electrodialysis (ED), nanofiltration (NF), forward osmosis (FO), and hybrid configurations that combine membrane operations with renewable-energy systems or thermoelectric assistance.

This review focuses on four major dimensions of analysis: system configuration, thermoelectric function, desalination performance, and techno-economic implications. The thermoelectric function is considered in terms of both electricity generation and localized heating/cooling support, depending on the configuration reported in the literature. The desalination performance is examined through indicators such as water productivity, water quality, desalination efficiency, and TEM output, while economic feasibility is discussed through reported cost metrics and comparative economic trends.

The main objective of this review is not to claim universal superiority of thermoelectric-assisted desalination systems over conventional alternatives, but rather to critically examine the current state of development, identify the most promising areas of application, and highlight the research gaps that must be addressed for practical deployment. Special consideration is given to the potential of thermoelectric coupling in membrane desalination systems, where opportunities exist for enhanced thermal control, improved renewable-energy integration, and better utilization of low-temperature heat sources.

## 2. Review Methodology

### 2.1. Review Scope and Literature Selection Strategy

This study was conducted as a narrative review aimed at synthesizing and critically discussing the published literature on thermoelectric-assisted desalination systems, with emphasis on membrane desalination and membrane-hybrid configurations. The literature survey was designed to capture studies addressing desalination technologies coupled with TEMs, TEGs, renewable-energy systems, hybrid energy sources, or waste-heat recovery concepts relevant to water production.

The relevant literature was identified through searches in major scientific databases, including Scopus, Web of Science, ScienceDirect, and Google Scholar, using combinations of keywords related to thermoelectric desalination, hybrid desalination, renewable-energy-driven desalination, membrane desalination, membrane distillation, reverse osmosis, electrodialysis, forward osmosis, and techno-economic analysis. Additional studies were identified through backward and forward reference tracking of highly relevant review papers and research articles. The search was focused primarily on publications from recent years while also including selected earlier foundational studies necessary for conceptual or technological background.

The initial search identified approximately 250–300 articles. These included experimental studies, simulation-based investigations, modeling studies, comparative analyses, and review articles that provide useful insight into the role of thermoelectric devices in desalination. Studies were considered relevant if they discussed at least one of the following aspects: integration of thermoelectric devices into desalination systems, performance enhancement of desalination using thermoelectric effects, renewable-energy coupling in membrane or hybrid desalination, or techno-economic assessment of thermoelectric-assisted desalination systems. Greater emphasis was placed on studies involving membrane processes or configurations that could be reasonably linked to membrane-based desalination pathways. Data from approximately 150 articles was gathered and analyzed in the form of graphical representations and critical discussion throughout this review.

Because this article is a narrative review, the literature selection process was qualitative rather than based on strict systematic screening criteria. Nevertheless, efforts were made to ensure balanced coverage of major desalination classes, key thermoelectric-integration strategies, and both performance-related and economic aspects of the field. Studies with clearer technical detail, stronger relevance to desalination performance, and greater significance for membrane process development were prioritized in the discussion.

### 2.2. Thematic Organization of the Reviewed Literature

The selected literature was organized thematically in order to provide a structured understanding of how thermoelectric devices are being incorporated into desalination systems and where the strongest opportunities for membrane-related applications may lie. The discussion is first organized according to the major classes of desalination technologies, including thermal, membrane-based, and emerging systems, to establish the broader technological background. This is followed by a discussion of renewable-energy-powered desalination systems, where direct and indirect renewable coupling strategies are examined in relation to desalination performance and energy utilization.

A dedicated performance-oriented analysis is then used to evaluate the reported effects of thermoelectric integration on water productivity, module output, water quality, desalination efficiency, and economic feasibility. Within this framework, attention is given to the different functional roles of thermoelectric devices, including electricity generation from temperature gradients, localized cooling, localized heating, and combined thermal-management support. The literature is also interpreted in light of membrane desalination requirements, particularly where thermal control, modular integration, and low-grade energy recovery are relevant.

Finally, the reviewed studies are assessed from a critical perspective to identify technical limitations, reporting inconsistencies, comparison difficulties, and future research opportunities. This thematic organization enables the review to retain a broad overview of thermoelectric-assisted desalination while maintaining a clear emphasis on membrane desalination relevance, which is central to the scope of the present work.

## 3. Desalination and Classification

**Figure 1:**
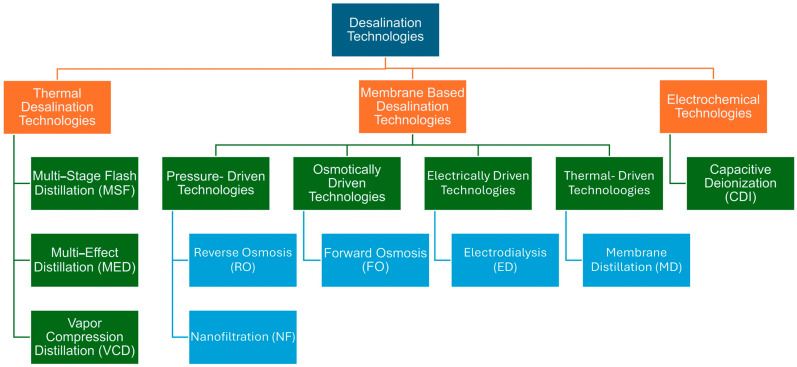
Broad classification of desalination technologies.

Desalination is the process of removing impurities and salts from saline or brackish water. The output is potable water, fit for human consumption. Desalination technologies can be categorized in a few different ways. According to one study, desalination technologies can be classified by working principle [22]. Another way to classify desalination technologies is to classify them based on the kind of input energy [23]. Classifications of desalination based on working principle and energy input are depicted in Figure S2 (Supplementary Information). Considering the above classifications, desalination technologies can be broadly categorized as shown in Figure 1.

### 3.1. Thermal Desalination Technologies

Thermal desalination (i.e., distillation) is one of the oldest technologies for treating saline water. The thermal energy required for boiling is produced in waste-heat boilers, in steam generators or by the extraction of back-pressure steam from turbines in power stations [24]. Some of the most common thermal desalination technologies are discussed below.

#### 3.1.1. Multi-Stage Flash Distillation (MSF)

MSF is a mature thermal process in which seawater is heated to a temperature of 90–120 °C and passed through 15–25 stages of chambers with progressively lower pressures causing flash evaporation in each stage [25]. MSF is an energy-intensive process, as it requires both thermal and mechanical energy [26]. The specific energy consumption of an MSF plant is 23–25 kWh·m^−3^ [27,28,29,30]. MSF, however, provides a high level of purification with effluent containing only 2–10 ppm TDS [31].

#### 3.1.2. Multi-Effect Distillation (MED)

The highest working temperature in an MED plant is 60–70 °C [32]. As a result, the overall energy consumption of MED is lower as compared to MSF. Brine is passed through 8–16 effects with progressively lower pressures and temperatures. Vapor generated from one effect serves as a heating source for the other effect. The specific energy consumption of a typical MED plant is 14–19 kWh·m^−3^ [33]. Aly et al. [34] integrated an absorption compressor into an MED system to reduce the overall energy consumption. The specific energy consumption of the system was 4.8 kWh·m^−3^. The calculated salt rejection of the system was 99.75%. The performance ratio (PR) of the MED-AB system is 3.8 which is 40% higher than the standalone MED system [34].

#### 3.1.3. Vapor Compression Distillation (VCD)

The VCD, also sometimes known as mechanical vapor compression (MVC), process may be used in combination with other processes, such as MED. The operating temperatures of these plants are low which reduces the degradation of tubes and vessels. VCD systems typically operate at temperatures of 70–90 °C and are usually suited for small- to medium-scale applications. The specific power consumption of VCD-powered MED systems can range from 10 kWh·m^−3^ to 13 kWh·m^−3^ for high-salinity water [35]. Ayber et al. [36] investigated a low-temperature MVC system and reported the specific energy consumption of a single-tube model as 11.5 kWh·m^−3^. Veza et al. [37] reported the energy consumption of VCD plants installed in Las Palmas, Spain, as 10.4–11.2 kWh·m^−3^.

### 3.2. Membrane Desalination Technologies

Membrane-based technologies use a semipermeable membrane to separate impurities and salts. Membrane-based methods such as reverse osmosis (RO) are replacing phase change desalination techniques. Some of the most common membrane-based desalination technologies are discussed below:

#### 3.2.1. Reverse Osmosis (RO)

The RO process is one of the most widely used membrane desalination methods. The advantage of RO systems is that they face fewer problems related to material corrosion as compared to MSF and MED. RO systems have high salt rejection rates of greater than 92% and a water recovery rate of 19.4–64% [38]. RO methods are also very energy-efficient as compared to thermal methods. The energy consumption of a typical RO system is 3–4 kWh·m^−3^ [39]. However, some of the disadvantages include clogging and scaling of the membrane.

#### 3.2.2. Electrodialysis (ED)

ED is an electrochemical process and has been widely investigated as an energy-efficient desalination technology. It uses both a membrane and an electric field to separate salts and ions from water [40]. In ED, alternating channels of concentrated brine and fresh water are created [41]. Two-stage ED has shown a salt removal efficiency of ~93%. The TDS on the inlet stream was 27 g·L^−1^ while the TDS at the end of stage 1 was 4.8 g·L^−1^ and at the end of stage 2 was 1.9 g·L^−1^, respectively [42]. The average energy consumption of the system was 3 kWh·m^−3^. Electrodialysis is also used in biochemical industries for the production of salt [43]. EDR can be used to treat rejected brine from the RO process. EDR has shown a cation and anion removal efficiency of 95% and can convert feed brine of TDS 29,300 mg·L^−1^ into water of TDS 425 mg·L^−1^ [44]. ED operates at low pressures compared to RO methods, which reduces pumping energy requirements. Thus, for low-salinity feedwater, ED is more energy-efficient compared to RO [45,46,47]. However, ED is only suitable for brackish water. In the case of seawater with a high concentration of dissolved solids, this process becomes highly unfeasible, as the current required is directly proportional to the amount of salt to be removed. Despite these limitations, ED has high potential for hybrid and resource recovery systems.

#### 3.2.3. Nanofiltration (NF)

NF is similar to the RO process. The only difference is that it uses a membrane that has relatively large pores. Thus, it only removes larger multivalent ions such as Mg^2+^ and Ca^2+^. The removal of monovalent ions is typically only 60–85% [48]. NF finds applications in wastewater treatment and the pharmaceutical industry [49]. It has limited applications in the desalination industry as it only removes some ions and lets some dissolved solids through. As NF requires less energy than RO, research is being carried out to make it feasible for seawater by introducing dual-stage units [49,50]. NF membranes with tight separating layers of polyamide (such as NF90) have relatively higher rejection rates for monovalent ions [51]. Zhou et al. [52] reported that ion exchange–nanofiltration (HIX–NF) desalination system could save 50% power consumption of that of RO. A dual-stage NF90-NF90 system had an energy consumption of 3.35 kWh·m^−3^ and the TDS of the desalinated water was 254 mg·L^−1^ [53]. A seawater desalination pilot system using a two-stage nanofiltration membrane (NF90) coupled with renewable energies can provide potable water at a cost of US $ 0.75 per m^3^ [54]. NF holds significant potential to be used as a pretreatment or hybrid component to reduce scaling, lower osmotic pressure requirements and enhance the overall efficiency of processes such as RO and FO. However, its performance as a standalone system is hindered due to the fact that the membranes used have larger pores, and so smaller and dissolved impurities cannot be removed. Thus, its performance is highly dependent on feedwater composition.

#### 3.2.4. Membrane Distillation (MD)

The membrane distillation method is a hybrid technique. It is both thermally driven and also uses a membrane. Saline water is converted into vapor and passed through a hydrophobic membrane onto a condensing surface. The membrane does not allow liquid water to pass through; thus, only the evaporated vapor passes through and condenses on the condensing surface, where it is collected as fresh water. The main advantage of MD is its simplicity. It requires a lower operating temperature (i.e., 30–90 °C) than conventional thermal processes, and a lower operating pressure (1 bar) than conventional membrane processes [55]. However, it requires more space and the overall energy consumption is the same as MSF and MED plants. The water recovery of MD plants is usually between 5 and 40% [56,57,58,59]; however, recovery of greater than 60% is possible [60]. The specific energy consumption of an MD process is roughly 17 kWh·m^−3^ or more, depending on system configuration [61]. This is more than that of MED plants and comparable to that of MSF plants. The MD process also requires that saline water be free from organic pollutants. All these limitations make it commercially less viable [62].

#### 3.2.5. Why Membrane Processes Are Especially Relevant for Thermoelectric Integration

Membrane desalination processes are especially relevant for thermoelectric integration because they combine modular process design with operating conditions that can benefit from improved thermal management, renewable-energy coupling, and localized recovery or utilization of low-grade energy. Compared with large conventional thermal desalination systems, membrane-based technologies are often better suited to decentralized deployment, flexible operation, and compact system design [63]. These characteristics make them attractive candidates for integration with thermoelectric devices in hybrid water treatment systems.

Among membrane processes, membrane distillation (MD) is particularly compatible with thermoelectric-assisted operation because it relies on a temperature difference across a hydrophobic membrane to drive vapor transport [64]. In such systems, thermoelectric devices may contribute by supporting local heating or cooling, stabilizing thermal gradients, or assisting in condensation enhancement, depending on the configuration. This creates opportunities for better use of solar energy, waste heat, or other low-temperature energy sources while maintaining the membrane-based character of the separation process.

Reverse osmosis (RO), electrodialysis (ED), and nanofiltration (NF) are primarily electrically driven processes, yet they may also benefit indirectly from thermoelectric integration in renewable-energy environments. For example, TEGs may recover part of the available heat from hybrid renewable systems or auxiliary thermal subsystems to support sensors, controls, small auxiliary loads, or other localized energy requirements [65]. Although the direct role of thermoelectrics in these electrically driven membrane systems is less obvious than in MD, the concept remains relevant where system efficiency, off-grid operation, or energy recovery are important design objectives.

From a broader perspective, membrane processes offer a promising platform for thermoelectric integration because of their scalability, process intensification potential, and increasing importance in sustainable desalination. Their relevance is further strengthened by the growing interest in hybrid systems that combine membranes with renewable-energy technologies, thermal-recovery schemes, and intelligent energy management. For these reasons, membrane desalination provides an important lens through which the value and limitations of thermoelectric-assisted desalination can be critically assessed.

Based on the presently available literature, the direct evidence for thermoelectric integration appears strongest for membrane distillation, more moderate and niche for forward osmosis and membrane capacitive deionization, and more indirect or application-specific for reverse osmosis, nanofiltration, and electrodialysis-related systems. Traisak et al. [66]. experimentally investigated the performance of a hybrid system by integrating direct contact membrane distillation (DCMD) with TEGs. The resulting system had a freshwater output of 8–9.5 kg·m^−2^ h^−1^ and an electrical output of 25–35 W·m^−2^. TEGs integrated with a membrane distillation system have also been shown to reduce heat exchange losses and in turn increase the performance of DCMD systems [67]. These outputs demonstrate the potential of TEG integration with a membrane-based desalination system.

### 3.3. Emerging Desalination Techniques

#### 3.3.1. Forward Osmosis (FO)

Forward osmosis is a process that has received more and more attention in recent years [68,69]. In an FO system, desalination is achieved by using an osmotic pressure gradient difference. Ezugbe et al. [70] investigated the performance of FO in treating municipal wastewater. The results of the study showed removal efficiency of 60.1 for chemical oxygen demand (COD), 100% for total suspended solids (TSS) and 98.36 for turbidity. The average pure-water flux was 5.62 L·m^−2^ h^−1^ across three sample runs. The energy consumption of a standalone FO system without draw solution reconcentration can range from 0.084 to 0.275 kWh·m^−3^ [71]. The advantage of an FO system is that the system itself is very energy-efficient as it does not require energy-hungry pumps to pass water through a membrane. Significant progress has been achieved recently in developing commercially viable FO membranes [72]. However, the drawback and limitation of an FO system is the energy required for draw solution regeneration. Of the total energy required by an FO system, 71–98% is used for draw solution regeneration [73]. Thus, the total specific energy consumption of an FO system can range from 1.4 to 25 kWh·m^−3^ depending on the energy required for draw solution regeneration [74].

#### 3.3.2. Capacitive Deionization (CDI)

Capacitive deionization is another upcoming desalination technology [75,76]. Similar to ED, in CDI, an electric field is produced between two carbon electrodes. As a result, the dissolved ions in seawater move towards the electrodes and are absorbed in the pores of the electrodes [77]. To increase the efficiency of this process, anion and cation membranes can be installed on electrodes to control the passing of ions into the pores. Such a process is called membrane capacitive deionization (MCDI) [78]. According to current research, CDI requires less energy and maintenance than ED. However, studies are still being carried out, as this is a relatively new technology [79,80]. The energy consumption of MCDI as compared to an RO system is shown in Figure S1 (Supplementary Information). NaCl solutions ranging from 10 mM to 90 mM are prepared and the performance of the MCDI system against the RO system is compared. In low concentrations of salt in feedwater (i.e., up to 30 mM), the MCDI system has lower energy consumption than an RO system [80]. Bales et al. [81] achieved a water recovery of 84% by using an MCDI system for water reuse application with a total energy consumption of 1.28 kWh·m^−3^ and treated water output of 1.0 m^3^·h^−1^.

#### 3.3.3. Other Emerging Non-Membrane Approaches

Secondary refrigerant freezing (SRF) is a very new desalination technique and is still currently under study and development. The SRF process is basically based on a liquid-to-solid phase transition [82]. SRF can achieve a salt removal efficiency of 98.5% after 8 freeze–melt cycles. The salinity of the water produced is only 610 mg·L^−1^ of the TDS. Even though the desalination rate is low, SRF only needs 15% of the energy needed in conventional thermal processes such as MSF [83].

Ion exchange resin (IXR) compounds are synthesized to react with ions within a solution. Alexandre Morel et al. [84] proposed a novel configuration for the use of IXR. The study proposed packing a microbial desalination cell (MDC) with IXR. The desalination rate of MDC packed with IXR was 1.5–8 times greater than that of conventional MDC.

In Gas Hydrate-based desalination (GHBD), hydrates are produced from host water molecules and gas guest molecules such as nitrogen, carbon dioxide, etc. [85]. Natural gas-based hydration can be used for desalination of water up to 160,000 mg·L^−1^ TDS and electrical conductivity (EC) of 173 ms·cm^−1^. The desalination efficiency of natural gas-based hydration ranges from 79.5 to 84.3% [86]. This technology has less energy requirements compared to MSF, but due to high capital costs, it has not been commercialized yet [87].

### 3.4. Energy Consumption of Desalination Processes

**Figure 2:**
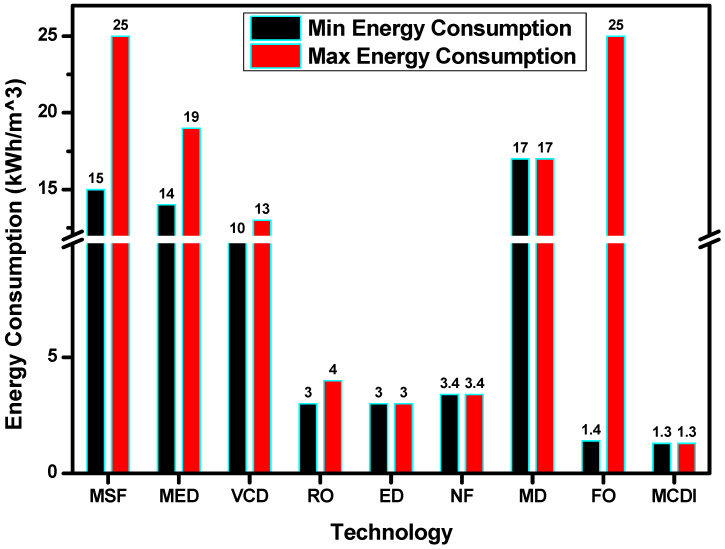
Energy consumption of different desalination technologies (trends based upon the results from studies [27,28,29,30,33,35,39,42,53,61,74,81]).

Specific energy consumption is one of the most important parameters while considering the feasibility of a desalination system for a specific location. Figure 2 shows the energy consumption of different desalination processes. Thermal processes such as MSF, MED and MD have the highest energy consumption. Of these thermal processes, MSF has the largest specific energy consumption of around 23–25 kWh·m^−3^. Thermal processes are recommended for areas that have cheap and abundant energy.

Membrane-based methods such as RO, ED and NF have relatively lower energy consumption in the range of 3–4 kWh·m^−3^. This makes membrane-based processes attractive for decentralized and renewable-assisted desalination. Membrane-based processes also work well in unison with renewable forms of energy because of their lower energy requirements. Emerging membrane-based technologies such as standalone FO without draw solution regeneration and MCDI have very low energy consumption of 0.3 and 1.28 kWh·m^−3^; however, their feasibilities and applications are still under study. The major contribution to energy consumption of an FO system is governed by the energy required for draw solution regeneration. This is the reason that the total energy consumption of an FO system can range from 1.4 to 25 kWh·m^−3^.

## 4. Renewable-Energy-Powered Desalination Systems

The depletion and environmental impact of fossil fuels highlight the need for renewable-powered desalination. Recent advancements in the field of renewable energy and energy storage have reduced costs and mitigated intermittency, making renewable-energy-based systems viable alternatives to fossil-fuel-based desalination. Desalination technologies based on renewable energy can be broadly classified into two categories: direct renewable-based desalination and indirect renewable-based desalination.

### 4.1. Direct Renewable-Based Desalination

In direct renewable-based desalination systems, renewable energy is used in its original form to run the desalination process. The most common example of direct solar desalination is the solar still. A conventional single-basin solar still without side insulation was 1.105 kg·m^−2^ d^−1^ and with side insulation was 2.84 kg·m^−2^ d^−1^ [88]. A double-basin solar still without side insulation has a productivity of 3.13 kg·m^−2^ d^−1^ and with side insulation has a productivity of 3.91 kg·m^−2^ d^−1^. The productivity of a triple-basin solar still was 12.635 kg·m^−2^ d^−1^. By increasing the number of basins, the productivity of a solar still increases. Geothermal energy could also be used to directly run desalination systems such as vacuum membrane distillation (VMD). Sarbatly et al. [89] experimented on a commercial VMD system which has a max water flux of 9.28 kg·m^−2^ h^−1^. The energy consumption of the system was 66.03 kW·kgh^−1^. Of the energy consumption, 95% energy consumption was thermal, which could be provided by warm geothermal water. The TDS of the water produced was 102 to 119 ppm. The VMD system powered by geothermal has a water production cost of 0.5 $·L^−1^ as compared to the water production cost of 1.22 $·L^−1^ for a standalone VMD system.

### 4.2. Indirect Renewable-Based Desalination

**Figure 3:**
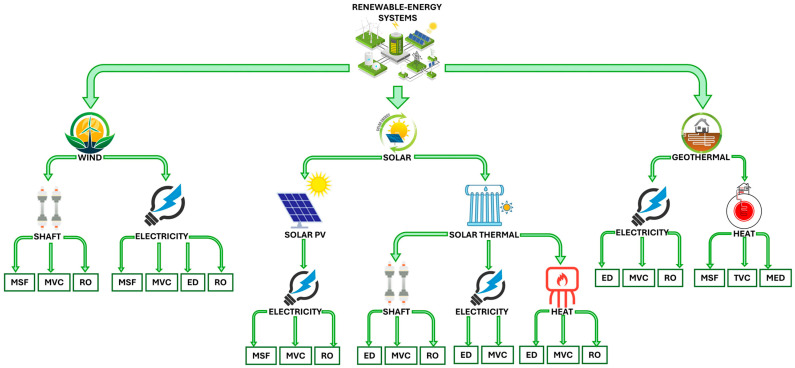
Possible couplings of renewable-energy systems with desalination systems (redrawn from [92]).

If renewable energy is first converted into electricity, which in turn is used to operate a desalination process, then such a system is called an indirect renewable-based desalination system. Kassem et al. [90] studied the performance of a hybrid SWRO plant powered by solar PVs, wind turbines, and a diesel generator. The system had an average power consumption of 2.7 kWh·m^−3^ and a recovery percentage of 44.7%. Alghoul et al. [91] designed a small test unit comprising a brackish water reverse osmosis (BWRO) system powered by a 2 kW PV system. Overall, 10 h of system use produced 5.1 m^3^ of fresh water with a TDS of less than 50 mg·L^−1^. The specific energy consumption of the system was 1.1 kWh·m^−3^. Different couplings of renewable-energy systems with common desalination technologies are shown in Figure 3 [92].

### 4.3. Renewable-Energy Coupling in Membrane Desalination Systems

The coupling of renewable energy with membrane desalination systems has emerged as a promising route toward sustainable and decentralized freshwater production. Membrane technologies, particularly reverse osmosis, membrane distillation, electrodialysis, and forward osmosis, are well suited for renewable-energy integration because of their modular nature, operational flexibility, and potential compatibility with intermittent or distributed power sources. In comparison with large centralized desalination facilities, renewable-driven membrane systems can offer greater adaptability for remote, off-grid, or small-scale applications where conventional energy infrastructure is limited [93].

Among these technologies, photovoltaic-driven reverse osmosis has received considerable attention due to the maturity of RO and the direct compatibility of photovoltaic electricity with electrically driven membrane systems. Wind-powered RO systems and hybrid photovoltaic–wind membrane desalination systems have also been investigated as means of improving energy reliability and reducing dependence on fossil fuels [94]. Similarly, electrodialysis and nanofiltration can be favorably coupled with renewable electric sources because their energy requirements are fundamentally electrical rather than thermal. In such configurations, renewable energy provides the primary driving force, while thermoelectric elements may serve supplementary roles related to local energy recovery or energy management.

Thermally driven membrane processes, especially membrane distillation, are particularly attractive in the context of renewable-energy coupling because they can utilize low-grade heat from solar thermal collectors, geothermal sources, or industrial waste heat. This makes membrane distillation a strong candidate for integration with thermoelectric devices, which can potentially exploit temperature gradients for electricity generation or assist thermal control at the module level. In hybrid renewable-energy systems, this creates opportunities for synergistic operation in which solar thermal input, photovoltaic electricity, and thermoelectric recovery are combined to improve overall system utility [95]. At present, the membrane desalination literature suggests that thermoelectric coupling is most mature in thermally driven membrane pathways such as membrane distillation, whereas in electrically driven membrane systems it is more often proposed for auxiliary energy recovery, localized thermal management, or hybrid system support rather than as a primary process driver.

**Figure 4:**
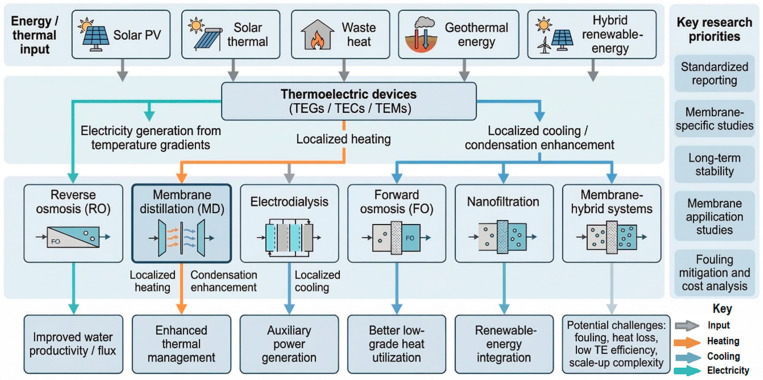
Conceptual pathways for thermoelectric integration in membrane desalination systems. Arrow colors indicate different thermoelectric functions: Teal/Green = electricity generation pathway; Orange = localized heating pathway; Blue = localized cooling/condensation enhancement pathway; Gray = general thermal input or auxiliary pathway.

The integration of renewable energy with membrane desalination is therefore not only an energy-supply strategy but also a process-design opportunity. It allows desalination systems to be developed as modular, hybridized platforms in which membrane separation, thermal recovery, and auxiliary electrical generation can operate in a coordinated manner. This is especially relevant for future membrane-hybrid systems seeking improved energy efficiency, lower environmental impact, and greater suitability for distributed water treatment applications. The major conceptual pathways through which thermoelectric devices may be integrated into membrane desalination systems, including their links with renewable-energy inputs, membrane process classes, and expected functional outcomes, are illustrated in Figure 4.

It should be noted that the integration pathways shown in Figure 4 represent potential strategies for the enhancement of desalination performance through integration with TEGs and renewable-energy sources. However, this integration is not always universally applicable and is dependent on system operating conditions and configuration. For example, in FO-based systems, the integration of TEGs and renewables is particularly advantageous when low-grade waste heat is available for the regeneration of draw. The benefit of this integration is also dependent on the development of energy-efficient regeneration techniques, as this step is a dominant factor contributing to the total specific energy consumption of an FO system.

Similarly, an RO system depends on a constant supply of electrical energy. Renewable sources such as solar and wind can effectively address the energy demands; however, due to their intermittent nature, electrical or thermal energy storage is required to ensure stable and continuous operation. The use of TEGs in RO systems is also limited due to their relatively low conversion efficiency and is therefore more suited to being a supplementary energy source rather than a primary one. Furthermore, the effectiveness of these hybrid pathways is influenced by factors including feedwater salinity, membrane fouling, system scale and the availability of renewable-energy supply. Therefore, these pathways should be considered as conditional integration strategies rather than universally optimal solutions. The successful implementation of these integrations is closely dependent on ongoing research in the fields of improved waste-heat recovery, advanced draw solution regeneration techniques, enhanced system energy efficiency and robust energy storage solutions.

## 5. Performance Analysis of Cogenerative/Hybrid Desalination Systems

**Figure 5:**
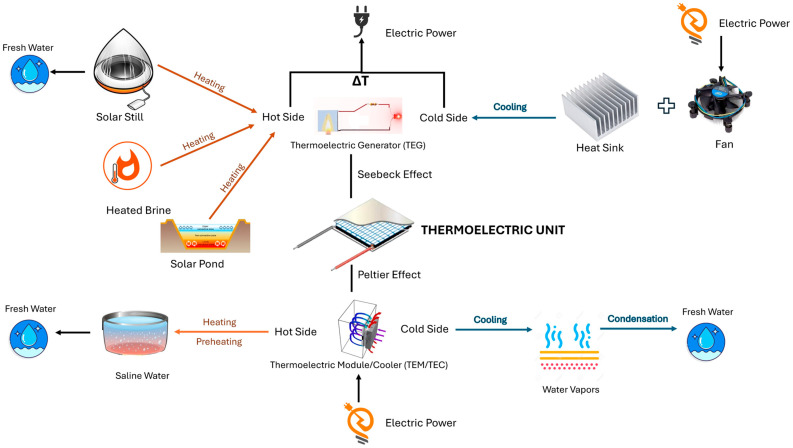
General schematic of TEG-incorporated hybrid desalination system.

A conventional desalination system has very high energy requirements. A cogenerative/hybrid desalination system incorporating a TEG can simultaneously generate electricity to somewhat increase overall efficiency. Similarly, a TEM can be used to either preheat the saline water or condense the heated vapor, thereby increasing the overall efficiency of the system by decreasing the energy requirements. A simplified schematic of such a system is shown in Figure 5.

The different factors that can be used to compare the performance of hybrid/regenerative systems are water output, TEG output, desalination efficiency and cost analysis.

### 5.1. Water Output

One of the main factors differentiating different desalination technologies/systems is the water output. However, the water output of a desalination system is inherently dependent on its scale. As such, direct comparisons between small-scale systems and large-scale plants do not yield any useful data. Furthermore, the actual water output of desalination systems may deviate from simulated estimates due to operational and environmental factors. Accordingly, this study aims to conduct a comparative analysis among small-scale desalination systems, as well as among large-scale systems, to ensure reliability and relevance in performance evaluation.

Shoeibi et al. [96] experimentally compared four different configurations of a solar-powered water desalination system. The daily water outputs for the four configurations were 6.67 mL·h^−1^, 6.56 mL·h^−1^, 6.79 mL·h^−1^ and 8.29 mL·h^−1^ respectively. The configuration incorporating mirror reflectors and thermal energy storage showed the most promising results as the reflector resulted in better solar intensity while thermal energy storage addressed the intermittent characteristics of solar energy. Date et al. [97] designed and manufactured a novel system using TEGs and heat pipes for the simultaneous generation of voltage and desalinated water. Their setup produced 64 g of water in 116 min of operation. This is equivalent to a daily water output of 33.1 mL·h^−1^. Saleque et al. [98] enhanced the performance of TEGs by covering the TEGs with rGO-coated cotton fabric. To compare the performance of the system, three configurations of the experimental setup were tested. The daily water outputs of the three system configurations were 11.35 mL·h^−1^, 32.7 mL·h^−1^ and 43.53 mL·h^−1^ respectively. The configuration with rGO coating and a heat sink had the best results because of the greater optical absorption characteristics of rGO-coated cotton fabric. Al-Kharabsheh et al. [99] proposed a water desalination process that uses passive vacuum generation to reduce the boiling point of water. Consequently, the latent heat of vaporization decreases, making it feasible to utilize low-grade heat sources. Due to the decreased pressure, the experimental setup achieved a water output of 108 mL·h^−1^.

Esfahani et al. [100] employed thermoelectric cooling in a small portable system to increase the condensation performance of the system. Due to the small size of the proposed system, the water output over the 9 days of experimentation was relatively low, i.e., 2 mL·h^−1^. Rahbar et al. [101] designed and built a portable solar still using thermoelectric cooling to condense the vapor and a heat pipe to improve the heat rejection from the hot side of the TEG. The water output for this system was 2.47 mL·h^−1^. The very low output highlights the limited practical scalability of such configurations despite their conceptual feasibility. Parsa et al. [102] employed both thermoelectric cooling and heating. Thermolelectric heating was used to preheat the saline water, while thermoelectric cooling was employed to condense the vapor. Nanofluid was used to further treat the water by removing harmful bacteria and pathogens. The water output for this system with and without an external condenser was 90.83 mL·h^−1^ and 58.75 mL·h^−1^, respectively. Despite the improved yield, system scalability and adoptability may be limited due to added system complexity. Shoeibi et al. [103] similarly suggested utilizing both sides of a TEM to improve the productivity of the solar still. The cool side is used to cool the glass cover of the solar still while the hot side is used to heat the saline water. In the study, the performance of the modified solar still was compared with that of a conventional solar still. It was observed that the daily water output of the modified solar still increased by 79.4%. The daily water output of the modified solar still was 16.33 mL·h^−1^ as compared to the daily water output of 9.16 mL·h^−1^ for the conventional solar still.

Maheswari et al. [104] used exhaust gas from a 5 hp diesel engine for desalination of saline water in a submerged horizontal tube straight pass evaporator (SHTE). The system had a water output of 1800 mL·h^−1^ without preheated saline water. In a variation of the same experiment the saline water was preheated to 60 C. The system under these conditions had a water output of 3000 mL·h^−1^. The system shows promise by utilizing waste heat from exhaust to produce potable water. Myneni et al. [105] proposed using a vacuum pump to decrease the pressure in the chamber so that low-grade heat could be used as the energy source. The daily water output for the proposed system was 310 mL·h^−1^. Shafii et al. [106] studied the effects of brine volume and forced convection on the water output of a solar still with evacuated tubes. When the tubes were half-filled with brine and operated without a fan, the system produced 485.44 mL·h^−1^, which increased to 636 mL·h^−1^ when fully filled. The incorporation of a fan further enhanced the yield to 728.16 mL·h^−1^. The experimental results highlight the increased system performance due to the effective heat exchange characteristics of forced convection. Gude et al. [107] used solar energy directly and indirectly for desalination. In the first phase of the experiment, direct solar energy was used to heat water directly inside an evaporation chamber. The daily water output was 206.25 mL·h^−1^. In subsequent experiments, initially a mirror reflector and finally a PV panel were added to the experimental setup. These modifications increased the intensity of input solar energy, thereby increasing the daily water output to 312.5 mL·h^−1^ and 500 mL·h^−1^ respectively.

**Figure 6:**
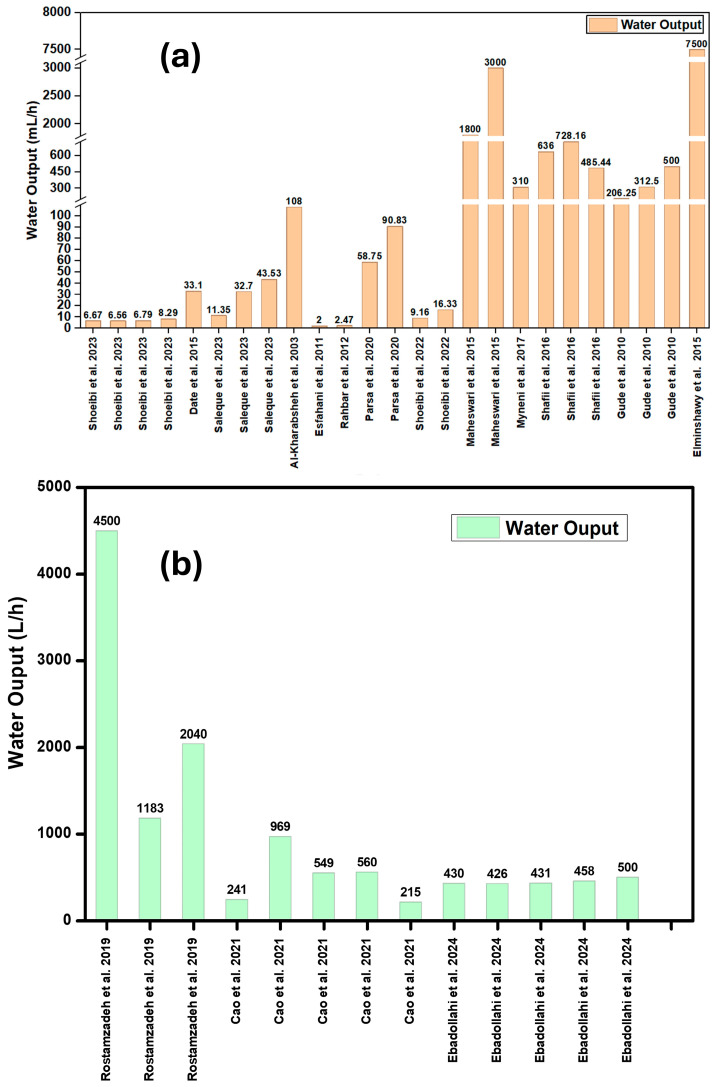
The estimated water output of (**a**) experimental (trends based upon the results from studies [96,97,98,99,100,101,102,103,104,105,106,107,108]) and (**b**) simulated (trends based upon the results from studies [109,110,111]) desalination setups.

Elminshawy et al. [108] developed a desalination system comprising an evacuated tube solar collector. The system had a daily water output of 7500 mL·h^−1^ at a temperature of 70 °C. The greater experimental output of the system may be attributed to system scale. Nevertheless, the system emphasized the importance of using waste gas emitted by thermal stations to drive a water desalination system coupled with a solar collector. The experimentally obtained water output of different proposed desalination systems is shown in Figure 6a and Table S1 (Supplementary Information). The water output varies significantly between different systems, because of many factors such as scale, efficiency, ambient temperature, etc.

Rostamzadeh et al. [109] investigated the utilization of salinity gradient solar ponds for harvesting thermal energy from solar radiation. The system also incorporated the use of TEGs to use the waste heat of discarded brine to produce electricity. They performed single-criterion optimization of the system and carried out parametric evaluation on an EES genetic algorithm (GA). The daily water output of different optimized systems, i.e., DWOD, ECGOTOD and CGOROD, was 4500 L·h^−1^, 1183 L·h^−1^ and 2040 L·h^−1^ respectively. A new layout for the generation of power and electricity was proposed by Cao et al. [110]. The study proposes a novel configuration for the cogeneration of electricity and distilled1 water by utilizing thermal energy stored in the lower convective zone (LCZ) of a salinity gradient solar pond (SGSP). The system also consists of two TEGs used to convert waste heat to electricity, which increases the overall efficiency of the system. Thermodynamic and thermo-economic tools are used for optimization and parametric study of the proposed system. The daily water outputs of the different system optimizations, i.e., base mode, TOM, EOM, COM and MOOM, are 241.1 L·h^−1^, 968.8 L·h^−1^, 549.4 L·h^−1^, 560.5 L·h^−1^ and 214.9 L·h^−1^ respectively.

Ebadollahi et al. [111] presented an innovative hybrid system. The study included thermodynamic and parametric evaluation of the system. The main source of energy was solar. However, solar energy was transferred to the system through a nanofluid. Syltherm 800 was used as the primary fluid. Different particles such as Cu, CuO, Al_2_O_3_ and TiO_2_ were infused with syltherm. The performance of different nanofluids was compared in terms of daily water output. Syltherm 800 alone had a daily water output of 430.6 L·h^−1^. Syltherm with different nano particles, i.e., Al_2_O_3_ nanoparticles, TiO_2_ nanoparticles, CuO nanoparticles and Cu nanoparticles, had water outputs of 425.8 L·h^−1^, 431.0 L·h^−1^, 458.4 L·h^−1^ and 499.7 L·h^−1^ respectively. Simulated water outputs of different proposed desalination systems are shown in Figure 6b.

### 5.2. Thermoelectric-Device Output

Thermoelectric devices can perform multiple functional roles in desalination systems depending on the system design, thermal boundary conditions and intended mode of operation. In the literature reviewed, these roles generally fall into four broad categories: electricity generation from temperature gradients, localized cooling, localized heating, and combined thermal-management support. A clear understanding of these functions is essential because the contribution of thermoelectric devices to desalination performance is strongly dependent on how they are integrated into the system.

In the first role, TEGs convert a temperature difference directly into electricity. Within desalination systems, this mode is typically associated with the recovery of low-grade thermal energy or the utilization of naturally occurring hot–cold-side gradients, such as those present in solar stills, solar ponds, condensers or hybrid thermal desalination units [112]. The electricity generated may not be sufficient to drive the entire desalination process, but it can contribute to auxiliary power demands, instrumentation or overall energy recovery. This function is particularly attractive in hybrid configurations where waste heat is available and would otherwise remain underutilized.

In the second and third roles, TEMs may be used for localized cooling or localized heating. Cooling functions are especially relevant where enhanced condensation is desired, whereas heating functions can support evaporation or feed preheating in thermally driven systems. In some configurations, both heating and cooling effects are exploited simultaneously to intensify the temperature gradient across the desalination unit. These applications are especially relevant to compact and experimental systems in which TEMs are used not only as energy-conversion devices but also as active thermal-management components.

The fourth role involves broader thermal-management support in hybrid desalination systems. In this case, thermoelectric devices may contribute indirectly by stabilizing operational conditions, redistributing thermal energy, or supporting integrated renewable-energy use [113]. This role is particularly relevant for membrane distillation and other membrane-hybrid systems where local temperature control can influence mass transfer, vapor flux, and overall process stability.

Because these roles differ significantly in mechanism and expected benefit, the literature should be interpreted with caution when comparing reported results. Studies using thermoelectric devices for waste-heat electricity generation are not directly comparable to those employing TEMs for active heating or cooling. With this contextual separation in mind, selected studies are discussed to highlight how thermoelectric devices have been applied in different desalination configurations.

Shoeibi et al. [96] connected two TEGs at the basin of a conventional solar still. The aim of the study was to increase the overall efficiency of the system. The study also explored the use of mirror reflectors and iron scalps as thermal storage to increase efficiency. The output of the three-system configuration was 2.13 W, 2.17 W and 2.5 W daily, respectively. Myneni et al. [105] used four TEGs to generate electricity. A temperature difference of 27 °C was achieved across the two ends of the TEG. This resulted in a power output of 0.94 W. Rostamzadeh et al. [109] proposed using two TEGs at different positions in the humidification–dehumidification (HDH) system. The TEGs used the waste heat of the distilled water and brine to generate electricity. Single-criterion optimization was carried out in the system. Exergy-based cogeneration gained-output ratio optimal design (ECGOROD) generated the highest average net electricity, i.e., 11.83 kW per month. Distilled water optimal design (DWOD) and cogeneration gained-output ratio optimal design (CGOROD) generated 3.16 kW and 9.11 kW of electricity per month.

Date et al. [97] designed the system to accommodate four TEGs. The heat is transferred to a set of heat pipes through the TEG. This creates a temperature difference of roughly 48 °C across the two ends of the TEGs. The TEGs used in the system have a power output of approximately 1.25 W when a temperature difference of 48 °C is maintained across the two ends. Cao et al. [110] conducted a thermodynamic and economic study of a proposed cogeneration system that simultaneously produces distilled water and electricity by employing two TEGs. Initially, a feasibility study of the base mode was carried out without carrying out any optimization. The TEGs had a net electricity output of 1.21 kW per month. Afterwards, multi-objective optimization was implemented. TEGs in thermal-optimal mode (TOM), exergy-optimal mode (EOM), cost-optimal mode (COM), and multi-objective-optimal mode (MOOM) had a net electricity output of 5.195 kW, 3.381 kW, 3.576 kW and 4.634 kW per month respectively. Common fluids such as water, ethylene glycol and other refrigerants play an important role in the refrigeration industry. However, their poor heat conductivity acts as a barrier to heat transfer which results in thermal losses [114]. To address this problem, Ebadollahi et al. [111] proposed using a nanofluid with suspended particles. Different nanoparticles were suspended in the nanofluid and the performance of the system was compared. A Syltherm base fluid infused with Cu nanoparticles showed the best results. Approximately 1.78 kW of power was produced by the TEG. The results of different parametric studies with different nanoparticles are shown in Table 1.

Saleque et al. [98] explored the use of rGO-coated cotton fabric to increase the evaporation efficiency of the system to 86.98%. To increase the overall efficiency of the system, 15 TEGs were also employed. Together they produced 339.6 mW of electricity and thus increased the system efficiency by 7.3%. Shafii et al. [106] experimentally investigated the effect of evacuated heat tubes in a novel solar still. The study employed the use of 20 TEGs. A temperature difference of 14.9 °C was maintained across the two ends of the TEGs. This temperature difference produced 1.32 W of power output. Specifications and details of the TEGs/TEMs integrated in different desalination setups are given in Table 1. TEGs/TEMs have been used in different capacities in different desalination systems. Esfahani et al. [100] and Rahbar et al. [101] have used TEMs/TECs for cooling in their experimental setups. Rahbar et al. [115] have also used TEM/TEC modules for heating. Parsa et al. [102] and Shoeibi et al. [103] have used TEMs/TECs for both heating and cooling in their experimental setups.

**Figure 7:**
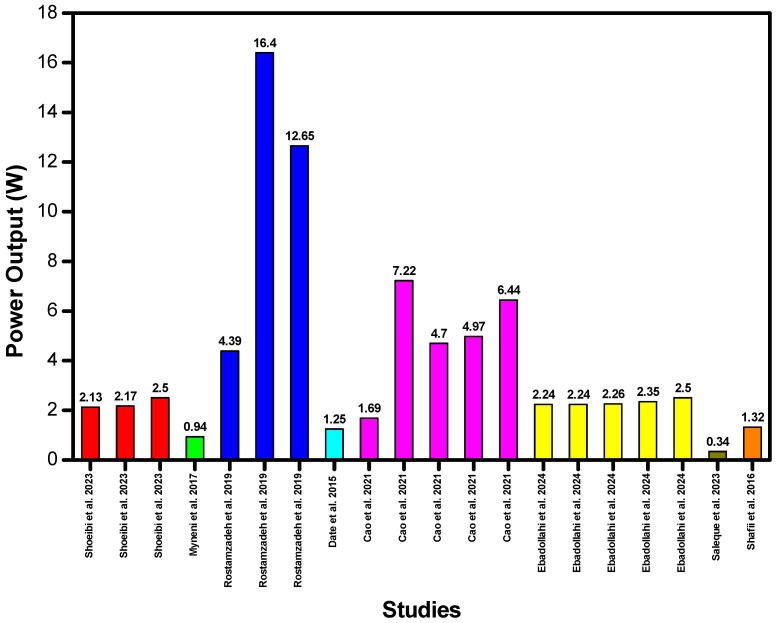
The power output of various TEG-integrated desalination setups (trends based upon the results from studies [96,97,98,105,106,109,110,111]).

Figure 7 presents the comparison of the power output of TEGs reported across different studies. Figure 7 suggests that exergy-based optimization, which focuses on maximizing the gained output ratio (GOR), produces the best results in terms of the energy output of the TEG, i.e., 16.4 W [109]. However, it should be noted that the output of a TEG depends on many factors such as system design, scale, number of TEGs, temperature difference achieved, the type of TEG used, etc. Also, some setups have used TEMs for heating and cooling applications that do not give a direct power output but indirectly increase the overall performance of the system. Cogeneration gained-output ratio optimal design (CGOROD) also produces a power output of 12.65 W, which signifies optimal system design. In contrast, the lowest recorded output of 0.339 W was attributed to the smaller scale and limited thermal gradient of the respective system. Notably, the integration of TEGs in this setup contributed to an overall efficiency improvement of 7.3%, highlighting the potential of TEGs to enhance energy utilization in solar–thermal desalination systems [98].

Even though the above studies have shown promising results, before integrating TEGs or TEMs in a system, certain design steps must be followed. To ensure maximum output, good thermal contact is essential. Thermal plasters or thermal paste, along with mechanical clamping, are required to ensure maximum heat transfer. The design of the heat sink is also crucial to maintaining a large ΔT across the two sides of the TEG/TEM. To handle fluctuating temperature inputs to the TEG, phase change material (PCM) or thermal mass can be used for heat transfer to ensure stable cooling/heating. Finally, the choice of TEG/TEM also plays an important role. The module should be chosen according to the required thermal performance, operating temperature range, module size, electrical characteristics and cost.

### 5.3. Water Quality/Desalination Efficiency

Only a few studies have reported the water inlet and outlet quality. Table 2 shows the TDS and pH (potential of Hydrogen) of water before and after desalination by different experimental setups. According to the WHO, a TDS below 300 is considered excellent for drinking; however, a TDS below 50 may indicate that water is tasteless and lacks important minerals [116]. The recommended pH range for potable water lies between 6.5 and 8.5. A pH value close to 7 is considered chemically neutral, whereas values exceeding 7.5 are indicative of a slightly alkaline character [116]. Shoeibi et al. [96] reported that the TDS and pH of the inlet water were 710 ppm and 7.87 respectively. A TDS of 710 ppm indicates that the water is not of good quality. The TDS of desalinated water was 136 ppm which indicates excellent quality drinking water. Esfahani et al. [100] reported a removal efficiency of 80%. The TDS of the inlet water was 598 ppm while the TDS of the outlet water was 123 ppm. Rahbar et al. [115] demonstrated effective desalination by reducing the TDS of water from 650.2 ppm to 92.8 ppm and achieving a desalination efficiency of 85%. The conventional and modified solar stills investigated by Shoeibi et al. [103] effectively reduced the TDS of saline water from 650 ppm to 120 ppm, demonstrating significant improvement in water quality through desalination.

**Figure 8:**
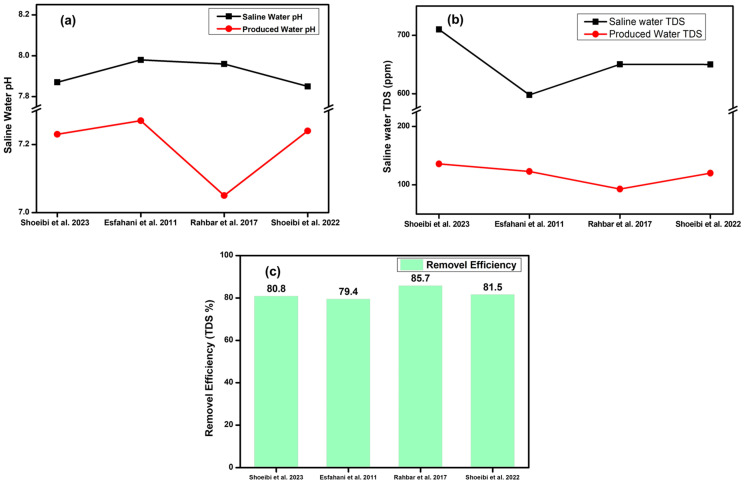
Desalination water quality parameters: (**a**) pH comparison, (**b**) TDS comparison, and (**c**) removal efficiency (trends based on the results from studies [96,100,103,115]).

Figure 8a shows the pH of water before and after desalination. Figure 8b shows the TDS of water before and after desalination. The removal efficiency of the desalination system can be calculated by the following equation:

$$\mathrm{Removal}\;\mathrm{Efficiency}\;=\;\frac{\mathrm{TDS}\left(\mathrm{before}\right)\;-\;\mathrm{TDS}\left(\mathrm{after}\right)}{\mathrm{TDS}\left(\mathrm{before}\right)}\times 100$$

The calculated removal efficiency of different desalination systems is shown in Figure 8c.

### 5.4. Cost Analysis

While designing a system for desalination, one of the most important factors governing feasibility is the cost. The different parameters that affect the cost are the capital recovery factor (CRF), sinking fund factor (SFF), fixed annual cost (FAC), annual salvage value (ASV), average annual productivity and annual cost. Considering the life of the system and all the above factors, the CPL (cost per liter) of the distilled water being collected by the desalination system can be calculated. Reported CPLs of the different proposed systems are given in Table S2 (Supplementary Information). The CPL of different experimental setups varies greatly, as the cost depends on many factors such as design, geographic location and system scale. The lowest CPL of water produced is the 0.00216 $·L^−1^ reported by Rahbar et al. [101], which was achieved by using thermoelectric cooling and heat pipes. This configuration significantly enhanced thermal management, evaporation and condensation efficiencies, leading to exceptionally low water production costs. Shafii et al. [106] used evacuated tubes for desalination. Evacuated tube collectors have high performance even in adverse climatic conditions. They trap the latent heat released during vapor condensation, which can be used to enhance the performance of the system. The experimental system had a low CPL of 0.00905 $·L^−1^. Elminshawy et al. [108] utilized solar energy coupled with low-grade waste heat, which resulted in lowering the CPL of the system to 0.014 $·L^−1^. The use of cheap low-grade heat along with solar energy resulted in low fuel costs. Shoeibi et al. [96] reported relatively higher costs which were due to the small scale of the experimental setup.

### 5.5. Limitations of Cross-Study Comparison

Although a wide range of studies have reported promising outcomes for thermoelectric-assisted desalination systems, direct comparison among published results remains difficult. The reviewed studies differ substantially in terms of desalination technology, operating principle, feedwater salinity, system scale, climatic conditions, heat-source characteristics, renewable-energy input, and performance metrics. As a result, numerical improvements reported in one system cannot always be generalized or directly compared with those obtained in another.

One major limitation arises from differences in scale. Many studies are based on laboratory-scale or proof-of-concept setups, whereas others involve modeled systems, pilot units, or application-oriented prototypes. The reported water productivity, thermoelectric output, and cost indicators can vary not only because of differences in technology but also because of differences in system size, boundary conditions, and experimental design. In addition, some studies present instantaneous performance values, while others report daily averages, seasonal productivity, or cumulative outputs, making consistent comparison even more challenging.

A second limitation concerns the inconsistency of reporting units and performance indicators. Water output may be expressed in mL·h^−1^, L·h^−1^, kg·m^−2^ d^−1^, or total daily production, while thermoelectric output may be reported as voltage, power, or conversion efficiency without standardized normalization. Similarly, cost analysis may be presented using different assumptions, currencies, scaling models, or component lifetimes. Such variation limits the reliability of direct quantitative comparison, particularly when studies are interpreted across different desalination classes.

A third challenge is the diversity of thermoelectric functions employed in the literature. Some studies use thermoelectric devices as electricity generators, others as thermal-management tools, and still others as multifunctional components. These distinct operational roles lead to different types of benefits and therefore should not be evaluated as if they represent a single uniform technology pathway. This issue is particularly important when comparing thermoelectric-assisted membrane systems with non-membrane systems or when discussing their practical relevance.

For these reasons, the findings summarized in this review should be interpreted as indicative of technological potential rather than as evidence of universally comparable performance gains. A more reliable basis for future comparison would require standardized reporting of operating conditions, normalized productivity metrics, membrane/process characteristics, thermoelectric function, and economic assumptions.

### 5.6. Implications for Membrane Desalination Design

The analysis presented till now in this section is primarily focused on conventional desalination systems to evaluate their performance characteristics, energy requirements and operational limitations. This analysis will provide a useful baseline for understanding different desalination approaches, particularly in terms of efficiency, water output, scalability and integration with waste heat or renewable-energy sources. However, despite their advantages, several constraints exist. Scalability, system complexity and limited adaptability are major hurdles to the implementation of these technologies. These observations highlight the need to explore alternative or complementary technologies that can address these challenges more effectively. In this context, membrane-based desalination systems have emerged as a dominant and promising class of technologies.

The reviewed literature suggests that thermoelectric integration may have meaningful implications for the design of future membrane desalination systems, particularly where compactness, renewable-energy utilization and thermal optimization are important. Although the strongest body of evidence currently comes from solar stills and other thermally driven proof-of-concept systems, several of the underlying principles are relevant to membrane-based desalination, especially membrane distillation and membrane-hybrid systems.

For membrane distillation, thermoelectric devices may be particularly valuable because the process depends strongly on maintaining an effective temperature gradient across the membrane. Localized heating or cooling provided by TEMs could potentially enhance evaporation and condensation conditions, stabilize thermal performance, and improve the use of low-grade thermal energy. In systems driven by solar thermal input or waste heat, TEGs may also provide a supplementary means of recovering part of the available temperature difference for auxiliary electrical use.

In electrically driven membrane processes such as reverse osmosis and electrodialysis, the role of thermoelectric integration may be less direct but still potentially beneficial in hybrid renewable systems. For example, thermoelectric elements may contribute to local energy recovery, support low-power components, or improve energy management in off-grid or decentralized units. However, the practical value of such integration in these processes remains insufficiently explored and requires more targeted membrane-specific investigation [117].

From a design perspective, the successful incorporation of thermoelectric devices into membrane systems will depend on careful consideration of module placement, heat-transfer pathways, membrane performance, fouling behavior, and overall energy balance. It is not sufficient to demonstrate that thermoelectric coupling can increase temperature gradients or generate auxiliary power under controlled conditions; the integration must also be compatible with stable long-term membrane operation and realistic system economics. Therefore, future work should move beyond proof-of-concept demonstrations toward membrane-oriented designs that evaluate performance durability, thermal efficiency, membrane integrity, and practical scalability.

To further clarify the membrane-specific relevance of thermoelectric integration, Table 3 summarizes the major membrane desalination and membrane-related processes in terms of their primary driving force, likely thermoelectric function, expected benefit, main technical limitations, present evidence level, and priority areas for future research. This process-level comparison highlights that thermoelectric integration is not equally relevant across all membrane systems. Rather, the strongest direct evidence currently appears in thermally driven membrane systems, particularly membrane distillation, whereas the roles in pressure-driven and electro-driven membrane processes are often more indirect, emerging, or application-specific.

## 6. Research Gaps, Challenges, and Future Directions for Membrane-Aligned Hybrid Desalination

Building on the comparative analysis of non-membrane desalination systems discussed in the previous section, this section focuses specifically on membrane-based desalination technologies. The current body of literature demonstrates that thermoelectric-assisted desalination is a promising but still developing field. While a variety of hybrid configurations have been proposed and tested, the available evidence remains fragmented across different desalination classes, experimental scales and thermoelectric operating modes. For membrane-aligned desalination systems in particular, the literature is still limited and does not yet provide a sufficiently consistent basis for broad conclusions regarding large-scale applicability or long-term commercial value. This section highlights the main technical and research gaps that should be addressed in order to advance thermoelectric integration in membrane desalination.

### 6.1. Technical Challenges

A major technical challenge in thermoelectric-assisted desalination lies in the inherently modest efficiency of thermoelectric devices under many practical operating conditions. Since thermoelectric performance depends strongly on maintaining a temperature gradient, the usefulness of such devices is constrained by heat-transfer limitations, thermal losses, and the difficulty of sustaining stable hot–cold-side conditions in compact desalination systems. In membrane-related applications, these challenges become even more important because any thermal intervention must be compatible with membrane stability, mass-transfer behavior, and long-term operational reliability [130].

Another challenge concerns system integration. The addition of thermoelectric devices introduces extra components, thermal interfaces, electrical connections, and control considerations that may complicate the overall system design. In membrane desalination systems, especially those intended for continuous or decentralized operation, the integration must not only improve performance but also preserve system simplicity and robustness. This is particularly important for membrane distillation, where thermal management can influence vapor flux and thermal efficiency, but poor integration may also increase heat losses or create localized operational instability.

Membrane fouling, scaling, wetting, and material durability are also relevant challenges that remain insufficiently addressed in the existing literature. Although many reported studies focus on short-term productivity improvements, fewer investigations consider how thermoelectric coupling may affect membrane condition over prolonged operation. For membrane-aligned hybrid desalination to advance beyond proof-of-concept systems, these practical limitations must be examined more systematically.

### 6.2. Reporting and Comparison Gaps

A recurring limitation in the reviewed literature is the lack of standardized reporting. Performance is often presented using different units, time scales, and evaluation criteria, which makes comparison across studies difficult. This problem is especially serious when water productivity, thermoelectric output, and cost are discussed together without consistent normalization. In several cases, studies report promising improvements, but the absence of detailed operating conditions, membrane properties, or system-scale information limits the interpretability of the results.

Another gap is the limited reporting of membrane-specific parameters in studies that could be relevant to membrane desalination. Important details such as membrane material, pore characteristics, module configuration, fouling tendency, salt rejection, long-term flux stability, and feedwater composition are often not reported in a form that enables meaningful comparison. This weakens the ability of the field to move from conceptual integration toward reproducible membrane process development.

There is also a need for clearer separation between different thermoelectric functions in the literature. Studies using TEGs for electricity generation should be reported and interpreted differently from those using TEMs for active cooling or heating. Without such distinction, the field risks combining fundamentally different performance mechanisms under a single label, which can create misleading impressions regarding technological maturity or practical benefit.

### 6.3. Opportunities for Membrane Desalination Systems

Despite the above challenges, important opportunities exist for the application of thermoelectric devices in membrane desalination systems. Among the various membrane processes, membrane distillation appears to offer the strongest near-term opportunity because of its dependence on thermal gradients and compatibility with low-grade heat [131]. TEMs may help intensify local temperature differences, improve condensation conditions, or support better utilization of hybrid renewable-energy inputs in membrane distillation systems.

There are also opportunities in renewable-powered membrane-hybrid systems designed for remote or off-grid applications. In such settings, the modular nature of membrane processes aligns well with the compactness of thermoelectric devices and the distributed character of solar or hybrid energy sources. Even where thermoelectric devices do not contribute large amounts of electricity, they may still add value through localized energy recovery, thermal balancing, or support for low-power auxiliary functions.

More broadly, thermoelectric integration may serve as part of a process-intensification strategy in advanced membrane systems. When combined with improved module design, better heat management, and optimized renewable-energy coupling, thermoelectric devices could contribute to more flexible and energy-aware desalination platforms. However, such opportunities will only be realized if future studies focus on membrane-specific design criteria rather than relying solely on generalized hybrid desalination concepts.

### 6.4. Future Research Priorities

Future research should prioritize membrane-specific studies that evaluate thermoelectric integration under realistic operating conditions. For membrane distillation in particular, there is a need for more systematic investigations of module-level thermal control, heat recovery, membrane wetting behavior, and long-term productivity under thermoelectric-assisted conditions. Studies should also explore whether the additional complexity introduced by thermoelectric components is justified by measurable gains in water flux, energy efficiency, or system reliability.

Standardization of reporting should be treated as a high priority. Future studies should report normalized productivity metrics, feedwater characteristics, membrane properties, system scale, thermoelectric operating mode, energy input, and cost assumptions in a consistent manner. This would substantially improve comparability and allow more reliable assessment of the actual value of thermoelectric coupling across different membrane processes.

Pilot-scale and long-duration studies are also required. Much of the current literature is based on short-term experiments or conceptual models, which are valuable for establishing feasibility but insufficient for judging real-world viability. The field would benefit from membrane-oriented demonstrations that include fouling analysis, durability testing, control strategy development, and techno-economic evaluation using clearly defined assumptions. Future priorities should also be differentiated by membrane process. For membrane distillation, the main need is module-level thermal design, long-term membrane wetting assessment, and scale-up of thermoelectric-assisted configurations. For reverse osmosis and nanofiltration, research should focus less on direct thermal integration and more on realistic techno-economic analysis, auxiliary energy recovery, and compatibility with existing high-efficiency energy recovery devices. In electrodialysis-related systems, forward osmosis, and MCDI/CDI, the priority lies in membrane-specific non-isothermal testing, stability and durability analysis, and better understanding of whether thermoelectric coupling provides a measurable advantage under continuous operating conditions.

Finally, future work should aim to move beyond broad hybrid-desalination claims and toward application-specific designs. Instead of asking whether thermoelectric devices are beneficial for desalination in general, future studies should identify where, how, and under what conditions thermoelectric integration provides a clear advantage within membrane desalination systems. This shift in focus will be essential for translating thermoelectric-assisted desalination from an interesting concept into a technically defensible and practically relevant membrane process strategy.

## 7. Recommendations

Thermoelectric integration represents a promising direction for improving the sustainability and functional versatility of desalination systems, particularly when combined with renewable-energy sources or low-grade heat. This review examined the role of thermoelectric devices across thermal, membrane-based and emerging desalination technologies, with particular emphasis on their relevance to membrane desalination. Thermoelectric devices can contribute to desalination through auxiliary electricity generation, localized heating or cooling, enhanced thermal management, and improved utilization of available temperature gradients.

Membrane-based systems, in particular membrane distillation, appear especially relevant for future thermoelectric integration because of their modularity, comparatively lower energy demands, compatibility with low-grade thermal energy, and suitability for hybrid and decentralized operation. Other membrane processes, such as reverse osmosis, electrodialysis, nanofiltration and forward osmosis, may also benefit indirectly from thermoelectric integration in renewable-powered or hybrid systems, although the available evidence for these applications remains more limited.

The reviewed studies indicate that thermoelectric-assisted desalination can improve water productivity, support better thermal utilization and in some configurations provide useful auxiliary power output. However, these benefits should be interpreted with caution because the current body of literature has significant variation in system design, operating conditions, feedwater characteristics, scale, reporting metrics and economic assumptions. As a result, direct comparison across studies remains difficult and broad claims regarding universal performance superiority or immediate large-scale applicability are not yet justified.

This review also highlights that the practical development of thermoelectric-assisted membrane desalination is still constrained by several challenges, including modest thermoelectric efficiency, heat-transfer limitations, integration complexity, insufficient long-term testing and limited membrane-specific performance reporting. In addition, many available studies remain at the laboratory or proof-of-concept scale, while pilot-scale demonstrations and standardized techno-economic evaluation are still lacking.

Overall, thermoelectric integration should be viewed as a context-dependent, valuable process intensification and not as a universally applicable solution for desalination, whose practical benefit depends strongly on system configuration and application context. The strongest near-term opportunities appear to lie in thermally driven membrane systems, particularly membrane distillation operating with renewable energy or low-grade heat. Future research should therefore focus on membrane-oriented system design, standardized reporting practices, long-duration testing and realistic scale-up studies to clarify where thermoelectric coupling can offer a technically meaningful and economically defensible advantage in sustainable desalination.

## Figures and Tables

**Table 1:** Performance comparison of thermoelectric devices in desalination systems.

Description of Setup	Thermoelectric Device Qty/Specs/Type	Thermoelectric Application	Thermoelectric Device Output	Reference
**Electric Power Generation by TEG**				
SS + TEG	02 × TEG1-12611 (56 × 56 mm)	TEG	Power output = 2.13 W	[96]
SS + TEG + iron scraps (thermal storage)			Power output = 2.17 W	
SS + TEG + iron scraps (thermal storage) + mirrors			Power output = 2.50 W	
TEG and passive vacuum desalination	-	TEG	Power output = 0.94 W	[105]
HDH system + solar pond (DWOD)	02 × Bismuth telluride (Bi2Te3) p-type TEG Figure of merit = 1.5	TEG	Power output = 4.39 W	[109]
HDH system + solar pond (ECGOROD)			Power output = 16.40 W	
HDH system + solar pond (CGOROD)			Power output = 12.65 W	
Evaporator vessel + TEG + heat pipe	04 × 127 thermoelectric couples Alumina Al_2_O_3_ 40 × 40 × 3 mm	TEG	Power output = 1.25 W	[97]
HDH system + Kalina Cycle + TEG + solar pond (base mode)	02 × Bismuth (III) Telluride Bi_2_Te_3_ p-type TEG	TEG	Power output = 1.69 W	[110]
HDH system + Kalina Cycle + TEG + solar pond (TOM)			Power output = 7.22 W	
HDH system + Kalina Cycle + TEG + solar pond (EOM)			Power output = 4.70 W	
HDH system + Kalina Cycle + TEG + solar pond (COM)			Power output = 4.97 W	
HDH system + Kalina Cycle + TEG + solar pond (MOOM)			Power output = 6.44 W	
Solar HDH system + TEG + Syltherm 800	01 × TEG coefficient = 4	TEG	Power output = 2.24 W	[111]
Solar HDH system + TEG + Syltherm 800 + AL_2_O_3_ nanoparticles			Power output = 2.24 W	
Solar HDH system + TEG + Syltherm 800 + TiO_2_ nanoparticles			Power output = 2.26 W	
Solar HDH system + TEG + + Syltherm 800 + CuO nanoparticles			Power output = 2.35 W	
Solar HDH system + TEG + Syltherm 800 + Cu nanoparticles			Power output = 2.50 W	
Solar desalination	15 × n-type bismuth telluride Bi2Te3 TEG p-type antimony telluride Sb2Te3 TEG	TEG	-	[98]
Solar desalination + rGO-coated cotton fabric			-	
Solar desalination + rGO-coated cotton fabric + heat sink			Power output = 0.34 W	
SS + evacuated tube(full)	20 × TEC1-12708 HB Corporation	TEG	Power output = 1.32 W	[106]
SS + evacuated tube(full)+ fan with TEG				
SS + evacuated tube (half full)				
**Heating/cooling by TEMs**				
Portable SS+ TEM (cooler)	TEC1-12706 HB Corporation	TEM/TEC	Cooling	[100]
Portable SS+ TEM (cooler) + heat pipe	TEC1-12708 HB Corporation	TEM/TEC	Cooling	[101]
Double-slope SS + TEM (heater)	04 × TEC1-12708 HB Corporation	TEM/TEC	Heating	[115]
SS + TEM (heater + cooler)	02 × TEC1-12708 HB Corporation for heating	TEM/TEC	Cooling and heating	[102]
SS (heater + cooler) + external condenser	02 × TEC1-12704 for cooling			
Modified SS + TEGs	-	TEM/TEC	Cooling and heating	[103]

**Table 2:** Water quality before and after desalination.

Saline Water Input pH	Saline Water Input TDS (ppm)	Freshwater Output pH	Freshwater Output TDS (ppm)	Removal Efficiency (TDS)	Reference
7.87	710	7.23	136	80.8	[96]
7.98	598	7.27	123	79.4	[100]
7.96	650.2	7.05	92.8	85.7	[115]
7.85	650	7.24	120	81.5	[103] (Conventional SS)
7.85	650	7.24	120	81.5	[103] (Modified SS with TEGs)

**Table 3:** Membrane-related opportunities and limitations for thermoelectric integration in desalination processes.

Process	Primary Driving Force	Potential Role of Thermoelectric Device	Expected Benefit	Main Technical Limitation	Current Evidence Level	Research Priority	Refs.
Reverse osmosis (RO)	Hydraulic pressure gradient (ΔP > Δπ)	Auxiliary power generation from external industrial waste heat to offset high-pressure pump loads; preheating feedwater via rejected heat.	Auxiliary power recovery; marginal flux enhancement due to reduced feedwater viscosity and increased diffusivity.	Low relevance for pressure-driven systems; extreme thermal sensitivity of thin-film composite polyamide layers; low thermoelectric efficiency vs. highly efficient isobaric energy recovery devices (ERDs).	Mostly indirect/conceptual evidence	Techno-economic analysis comparing TEG integration against modern isobaric ERDs; assessment of long-term thermal degradation on polyamide rejection.	[118,119]
Electrodialysis/electrodialysis reversal (ED/EDR)	Electric potential gradient (ΔE)	Imposing precise transmembrane thermal gradients using TEMs to induce thermophoretic ion mobility (the Soret effect).	Mitigation of concentration polarization (CP) at the boundary layer; significantly enhanced output power density in reverse electrodialysis (RED).	Extreme integration complexity; difficulty maintaining stable cross-membrane temperature differentials; parasitic heat conduction negating thermodynamic gains.	Emerging/sparse evidence	Membrane-specific experiments under non-isothermal conditions; long-term performance testing of ion-exchange resins under sustained thermal gradients.	[119,120,121]
Nanofiltration (NF)	Hydraulic pressure gradient (ΔP) combined with Donnan steric exclusion	Purely auxiliary power for feed pumps within complex polygeneration flowsheets; localized thermal management for upstream brine concentration.	Viscosity reduction; seamless integration into multi-stage zero liquid discharge (ZLD) or critical mineral (e.g., lithium) recovery architectures.	Minimal direct relevance for pressure-driven sieving; thermal stress causing flux dispersion; high risk of scaling at elevated temperatures.	Mostly indirect/conceptual evidence	Pilot-scale validation within integrated modular flowsheets (e.g., G-TEG systems); scaling mitigation and pretreatment optimization studies.	[122,123,124]
Membrane distillation (MD/VMD/DCMD/AGMD)	Transmembrane vapor pressure gradient (ΔP*_v_*) induced by a thermal gradient (ΔT)	TEGs for simultaneous heat recovery and electricity generation; TECs for internal pumping of the latent heat of condensation back to the feed.	Unprecedented process intensification; highly efficient self-contained heating/cooling loops; combined generation of fresh water and auxiliary electricity.	Severe parasitic heat losses; low thermoelectric figure of merit (*ZT*) limiting the overall gain output ratio (GOR); thermal contact resistance.	Strong direct evidence	Module-level thermal design to minimize contact resistance; scaling up electrically powered self-contained MD concepts; long-term membrane wetting studies.	[66,119,125]
Forward osmosis (FO)	Osmotic pressure gradient (Δπ)	Localized, highly controlled heating and cooling for the regeneration of thermo-responsive draw solutes (TRDS) exhibiting lower critical solution temperatures (LCST).	Energy-efficient draw solution recovery via liquid–liquid phase separation without the massive enthalpy penalty of bulk water boiling; improved renewable integration.	Weak thermal gradients at scale; high integration complexity for continuous phase separation; reverse solute flux of ionic liquids.	Moderate direct evidence	Draw solute-specific thermal design; fouling, toxicity, and durability studies of ionic liquids; continuous regeneration loop engineering.	[126,127]
Membrane capacitive deionization (MCDI/CDI)	Capacitive/electrochemical ion electrosorption into the electric double layer (EDL)	Thermal energy harvesting utilizing the fundamental temperature dependence of the electric double-layer capacitance during intermittent cycles.	Improved overall energy utilization; reduced specific electrical energy consumption (SEC) via precise temperature-swing discharge cycles.	Weak overall thermodynamic energy recovery (sub-1% Carnot efficiency); thermal degradation of carbonaceous electrodes and ion-exchange membranes over multiple cycles.	Limited direct evidence	Optimization of synchronized thermal and electrical cycles; long-term capacitive cycling stability; advanced thermal insulation of electrochemical cells.	[128,129]

## Data Availability

The raw data supporting the conclusions of this article will be made available by the authors on request.

## Supplementary Materials

The following supporting information can be downloaded at https://www.mdpi.com/article/10.3390/membranes16050175/s1, Figure S1: Energy Consumption—MCDI vs RO; Figure S2: Classification of desalination; Table S1: Water output of hybrid/co-generative systems; Table S2: Cost Per Liter (CPL).
