# Supplementary material for: Thermoelectric Generators (TEGs) and Renewable-Energy-Integrated Membrane-Based Hybrid Desalination Systems
Source: Membranes (Basel). 2026 May 13;16(5):175. doi: 10.3390/membranes16050175 (PMC13209115; doi:10.3390/membranes16050175)
Supplement: Supplementary file 1 [file membranes-16-00175-s001.zip › membranes-4247939-supplementary.pdf]

Thermoelectric Generators (TEGs) and Renewable-Energy-Integrated Membrane-Based Hybrid Desalination Systems

M. Hamza Asif Awan [1], Ashraf Aly Hassan [2], Asad Ali Zaidi [3,4] and Muhammad Asad Javed [1,*]

[1] Department of Mechanical Engineering, School of Mechanical & Manufacturing Engineering (SMME),
National University of Sciences and Technology (NUST), H-12, Islamabad 44000, Pakistan; mawan.me23smme@student.nust.edu.pk

[2] Department of Civil and Environmental Engineering, United Arab Emirates University, Al Ain 15551, United Arab Emirates; alyhassan@uaeu.ac.ae

[3] Department of Mechanical Engineering, Faculty of Engineering, Islamic University of Madinah, Medina 42351, Saudi Arabia; sali@iu.edu.sa

[4] Sustainability Research Center, Islamic University of Madinah, Medina 42351, Saudi Arabia

* Correspondence: asad.javed@smme.nust.edu.pk; Tel.: +92-51-90856066

Figure S1

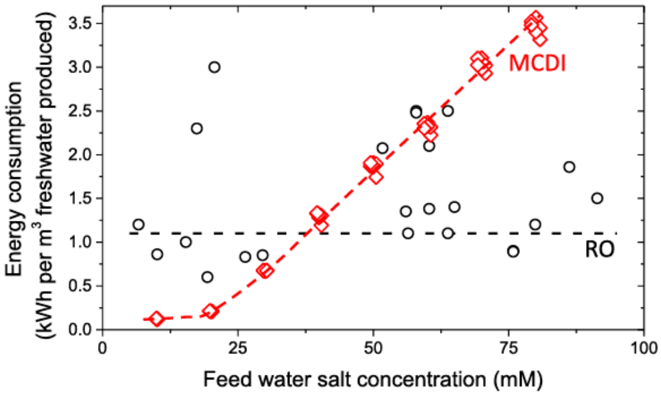

Figure S1. Energy Consumption - MCDI vs RO [1]

Figure S2

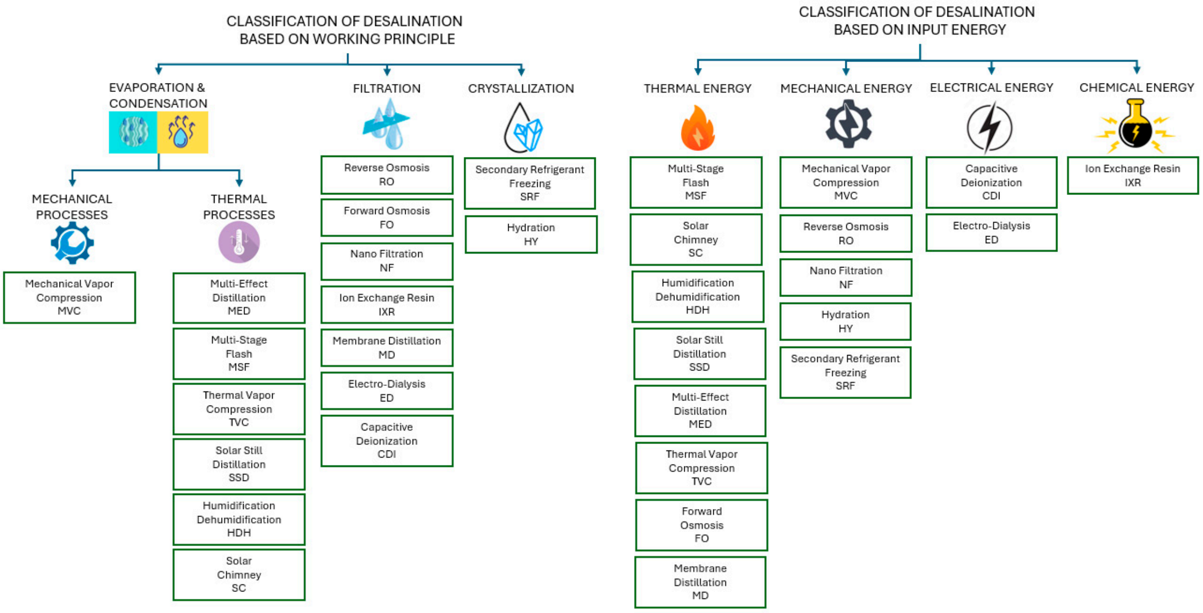

Figure S2. Classification of desalination [2]

Table S1

Table S1. Water output of hybrid/co-generative systems

Description	Fresh water Output	Reference
**Experimental Systems**		
SS	6.67 mL h^{-1}	[3]
SS + TEG	6.56 mL h^{-1}	
SS + TEG + Iron scraps (thermal storage)	6.79 mL h^{-1}	
SS + TEG + Iron scraps (thermal storage) + mirrors	8.29 mL h^{-1}	
Evaporator Vessel + TEG + Heat pipe	33.10 mL h^{-1}	[4]
Solar Desalination	11.35 mL h^{-1}	[5]
Solar Desalination + rGO coated cotton fabric	32.70 mL h^{-1}	
Solar Desalination + rGO coated cotton fabric +heat sink	43.53 mL h^{-1}	
Solar Heating + evaporator + condenser	108 mL h^{-1}	[6]
Portable SS+ TEM (cooler)	2 mL h^{-1}	[7]
Portable SS+ TEM (cooler)+ Heat pipe	2.47 mL h^{-1}	[8]
SS + TEM (heater + cooler)	58.75 mL h^{-1}	[9]
SS (heater + cooler) + External condenser	90.83 mL h^{-1}	
Conventional SS	9.16 mL h^{-1}	[10]
Modified SS + TEGs	16.33 mL h^{-1}	
SHTE + diesel engine exhaust	1800 mL h^{-1}	[11]
SHTE + diesel engine exhaust + preheated saline	3000 mL h^{-1}	
TEG and Passive Vacuum Desalination	310 mL h^{-1}	[12]

SS + Evacuated tube(full)	636 mL h⁻¹	[13]
SS + Evacuated tube(full)+ fan with TEG	728.16 mL h⁻¹	
SS + Evacuated tube (half full)	485.44 mL h⁻¹	
Direct Solar Energy + Evaporation chamber + Heat exchanger	206.25 mL h⁻¹	[14]
Direct Solar Energy + Evaporation chamber + Heat exchanger+ Reflector	312.5 mL h⁻¹	
Direct Solar Energy + PV panel + Heat exchanger+ Evaporation chamber	500 mL h⁻¹	
Desalination system + Solar energy + low grade waste heat	7500 mL h⁻¹	[15]
**Simulated systems**		
HDH system + Solar pond (DWOD)	4500 L h⁻¹	[16]
HDH system + Solar pond (ECGOROD)	1183 L h⁻¹	
HDH system + Solar pond (CGOROD)	2040 L h⁻¹	
HDH system + Kalina Cycle + TEG + Solar Pond (Base Mode)	241 L h⁻¹	[17]
HDH system + Kalina Cycle + TEG + Solar Pond (TOM)	969 L h⁻¹	
HDH system + Kalina Cycle + TEG + Solar Pond (EOM)	549 L h⁻¹	
HDH system + Kalina Cycle + TEG + Solar Pond (COM)	560 L h⁻¹	

HDH system + Kalina Cycle + TEG + Solar Pond (MOOM)	215 L h^{-1}	
Solar HDH system + TEG + Syltherm 800	430 L h^{-1}	[18]
Solar HDH system + TEG + Syltherm 800+AL2O3 nanoparticles	426 L h^{-1}	
Solar HDH system + TEG + Syltherm 800+TiO2 nanoparticles	431 L h^{-1}	
Solar HDH system + TEG + +Syltherm 800+CuO nanoparticles	458 L h^{-1}	
Solar HDH system + TEG + Syltherm 800+Cu nanoparticles	500 L h^{-1}	

Table S2

Table S2. Cost Per Liter (CPL)

Description	Cost Per Litre (CPL) $/L	Reference
SS	0.062	[3]
SS + TEGs	0.075	
SS + TEGs + Iron scraps (thermal storage)	0.074	
SS + TEGs + Iron scraps (thermal storage) + mirrors	0.071	
Portable SS+ TEMs/TECs (cooler)	0.00312	[7]
Portable SS+ TEMs/TECs (cooler)+ Heat pipe	0.00216	[8]
SS + evacuated tube collectors + TEMs/TECs	0.00905	[13]
Double slope SS + TEMs/TECs	0.0569	[19]
Desalination system + Solar energy + low grade waste heat	0.014	[15]
Conventional SS	0.0287	[10]
SS + TEGs	0.0268	

Bibliography'

[1] S. Porada, R. Zhao, A. Van Der Wal, V. Presser, and P. M. Biesheuvel, "Review on the science and technology of water desalination by capacitive deionization," *Prog Mater Sci*, vol. 58, no. 8, pp. 1388–1442, Oct. 2013, doi: 10.1016/J.PMATSCI.2013.03.005.

[2] D. Curto, V. Franzitta, and A. Guercio, "A Review of the Water Desalination Technologies," *Applied Sciences 2021, Vol. 11, Page 670*, vol. 11, no. 2, p. 670, Jan. 2021, doi: 10.3390/APP11020670.

[3] S. Shoeibi, M. Saemian, S. M. Parsa, M. Khiadani, S. A. A. Mirjalily, and H. Kargarsharifabad, "A novel solar desalination system equipped with thermoelectric generator, reflectors and low-cost sensible energy-storage for co-production of power and drinking water," *Desalination*, vol. 567, p. 116955, Dec. 2023, doi: 10.1016/J.DESAL.2023.116955.

[4] A. Date, L. Gauci, R. Chan, and A. Date, "Performance review of a novel combined thermoelectric power generation and water desalination system," *Renew Energy*, vol. 83, pp. 256–269, Nov. 2015, doi: 10.1016/J.RENENE.2015.04.024.

[5] A. M. Saleque *et al.*, "rGO coated cotton fabric and thermoelectric module arrays for efficient solar desalination and electricity generation," *J Mater Chem A Mater*, vol. 12, no. 1, pp. 405–418, Dec. 2023, doi: 10.1039/D3TA04715F.

[6] S. Al-Kharabsheh and D. Y. Goswami, "Experimental study of an innovative solar water desalination system utilizing a passive vacuum technique," *Solar Energy*, vol. 75, no. 5, pp. 395–401, Nov. 2003, doi: 10.1016/J.SOLENER.2003.08.031.

[7] J. A. Esfahani, N. Rahbar, and M. Lavvaf, "Utilization of thermoelectric cooling in a portable active solar still — An experimental study on winter days," *Desalination*, vol. 269, no. 1–3, pp. 198–205, Mar. 2011, doi: 10.1016/J.DESAL.2010.10.062.

[8] N. Rahbar and J. A. Esfahani, "Experimental study of a novel portable solar still by utilizing the heatpipe and thermoelectric module," *Desalination*, vol. 284, pp. 55–61, Jan. 2012, doi: 10.1016/J.DESAL.2011.08.036.

[9] S. M. Parsa, A. Rahbar, M. H. Koleini, S. Aberoumand, M. Afrand, and M. Amidpour, "A renewable energy-driven thermoelectric-utilized solar still with external condenser loaded by silver/nanofluid for simultaneously water disinfection and desalination," *Desalination*, vol. 480, p. 114354, Apr. 2020, doi: 10.1016/J.DESAL.2020.114354.

[10] S. Shoeibi, N. Rahbar, A. Abedini Esfahlani, and H. Kargarsharifabad, "Energy matrices, economic and environmental analysis of thermoelectric solar desalination using cooling fan," *J Therm Anal Calorim*, vol. 147, no. 17, pp. 9645–9660, Sep. 2022, doi: 10.1007/S10973-022-11217-7/METRICS.

[11] K. S. Maheswari, K. Kalidasa Murugavel, and G. Esakkimuthu, "Thermal desalination using diesel engine exhaust waste heat — An experimental analysis," *Desalination*, vol. 358, pp. 94–100, Feb. 2015, doi: 10.1016/J.DESAL.2014.12.023.

[12] N. S. Myneni, A. Date, M. Ward, P. Gokhale, and M. Gay, "Combined Thermoelectric Power Generation and Passive Vacuum Desalination," *Energy Procedia*, vol. 110, pp. 262–267, Mar. 2017, doi: 10.1016/J.EGYPRO.2017.03.137.

[13] M. B. Shafii, M. Shahmohamadi, M. Faegh, and H. Sadrhosseini, “Examination of a novel solar still equipped with evacuated tube collectors and thermoelectric modules,” *Desalination*, vol. 382, pp. 21–27, Mar. 2016, doi: 10.1016/J.DESAL.2015.12.019.

[14] V. G. Gude and N. Nirmalakhandan, “Sustainable desalination using solar energy,” *Energy Convers Manag*, vol. 51, no. 11, pp. 2245–2251, Nov. 2010, doi: 10.1016/J.ENCONMAN.2010.03.019.

[15] N. A. S. Elminshawy, F. R. Siddiqui, and G. I. Sultan, “Development of a desalination system driven by solar energy and low grade waste heat,” *Energy Convers Manag*, vol. 103, pp. 28–35, Oct. 2015, doi: 10.1016/J.ENCONMAN.2015.06.035.

[16] H. Rostamzadeh, A. S. Namin, P. Nourani, M. Amidpour, and H. Ghaebi, “Feasibility investigation of a humidification-dehumidification (HDH) desalination system with thermoelectric generator operated by a salinity-gradient solar pond,” *Desalination*, vol. 462, pp. 1–18, Jul. 2019, doi: 10.1016/J.DESAL.2019.04.001.

[17] Y. Cao, H. A. Dhahad, T. Parikhani, A. E. Anqi, and A. M. Mohamed, “Thermo-economic evaluation of a combined Kalina cycle and humidification-dehumidification (HDH) desalination system integrated with thermoelectric generator and solar pond,” *Int J Heat Mass Transf*, vol. 168, p. 120844, Apr. 2021, doi: 10.1016/J.IJHEATMASSTRANSFER.2020.120844.

[18] M. Ebadollahi, B. Shahbazi, and H. Ghaebi, “Efficiency and flexibility enhancement of nanofluid-based hybrid solar desalination system equipped with thermoelectric generator for eco-friendly freshwater and power cogeneration,” *Process Safety and Environmental Protection*, vol. 190, pp. 108–122, Oct. 2024, doi: 10.1016/J.PSEP.2024.07.077.

[19] N. Rahbar, A. Gharaiian, and S. Rashidi, “Exergy and economic analysis for a double slope solar still equipped by thermoelectric heating modules - an experimental investigation,” *Desalination*, vol. 420, pp. 106–113, Oct. 2017, doi: 10.1016/J.DESAL.2017.07.005.
